# Gapless genome assembly of *Fusarium verticillioides*, a filamentous fungus threatening plant and human health

**DOI:** 10.1038/s41597-023-02145-8

**Published:** 2023-04-20

**Authors:** Gang Yao, Weikai Chen, Jie Sun, Xiangfeng Wang, Huan Wang, Tan Meng, Lili Zhang, Li Guo

**Affiliations:** 1 Peking University Institute of Advanced Agricultural Sciences, Shandong Laboratory of Advanced Agricultural Sciences in Weifang, Weifang, Shandong 261325 China; 2 Weifang Institute of Technology, College of Modern Agriculture and Environment, Weifang, Shandong 262500 China; 3 Present Address: China Agricultural University, College of Information and Electrical Engineering, Beijing, 100091 China

**Keywords:** Fungal genomics, Sequence annotation, Fungal genetics

## Abstract

*Fusarium verticillioides* is a filamentous fungus that causes plant diseases and harms human health through cancer-inducing mycotoxin and life-threatening Fusariosis. Given its threat to agriculture and public health, genome assembly of this fungus is critical to our understanding of its pathobiology and developing antifungal drugs. Here, we report a gap-free genome assembly of *F. verticillioides* using PacBio HiFi data and high-throughput chromosome capture (Hi-C) sequencing data. The assembled 42.0 Mb sequence contains eleven gapless chromosomes capturing all centromeres and 19 of all 22 telomeres. This assembly represents a significant improvement over previous version on contiguity (contig N50: 4.3 Mb), completeness (BUSCO score: 99.0%) and correctness (QV: 88.8). A total of 15,230 protein-coding genes were predicted, 6.2% of which are newly annotated genes. In addition, we identified three-dimension chromatin structures such as TADs-like structures and chromatin loops based on Hi-C data of ultra-high coverage. This gap-free genome of *F. verticillioides* is an excellent resource for further panoramic understanding mechanisms of fungal genome evolution, mycotoxin production and pathogenesis on plant and human host.

## Background & Summary

*Fusarium verticillioides*, a filamentous fungus belonging to *Fusarium fujikuroi* species complex, causes Fusarium ear rot of maize, a major crop worldwide. Besides yield loss, various mycotoxins are produced during fungal infection of maize, reducing the quality of corn products. The best characterized *F. verticillioides* mycotoxins are fumonisins, a group of polyketide mycotoxins associated with esophageal cancer and neural tube birth defects in human populations consuming the contaminated maize products^1^. Although *F. veticillioides* is considered nonpathogenic to healthy human being, it can become a serious threat to individuals with compromised immune system such as those infected by undergoing organ transplants^2^. Human infection by *F. verticillioides* commonly known as Fusariosis has been a surging life threat to the immunocompromised patients due to limited options of antifungal drugs for treatment and emergence of multi-drug resistance^3^. Therefore, elucidation of molecular mechanisms underlying fungal pathogenesis and antifungal resistance in *F. verticillioides* is crucial to both agricultural safety and public health.

Despite the importance of this fungus, its complete genome sequence has not been assembled and thoroughly analyzed, impeding dissection of molecular and evolutionary mechanisms underlying its pathogenesis, secondary metabolism and drug resistance. The first genome assembly of *F. verticillioides* strain 7600 was released in 2010^4^ with a contig N50 of 392.3 kb. Recently, several updated versions of *F. verticillioides* genome assemblies are available in NCBI (National Center for Biotechnology Information) genome database. Although these genome assemblies have since facilitated the genetic studies of fungal biological processes, they are highly fragmented with several hundreds to thousands of contigs. The fact that *F. verticillioides* has 11 chromosomes suggests the presence of gaps in these assembly versions. Furthermore, no telomere and centromere sequences have been reported in any *F. verticillioides* genome assembly available, leaving these essential and complex genomic regions unexplored. A complete genome sequence for *F. verticillioides* would enable accurate characterization of the fungal genome function, regulation and evolution, shedding light on mechanisms of growth, development, pathogenicity and mycotoxin production.

**Fig. 1:**
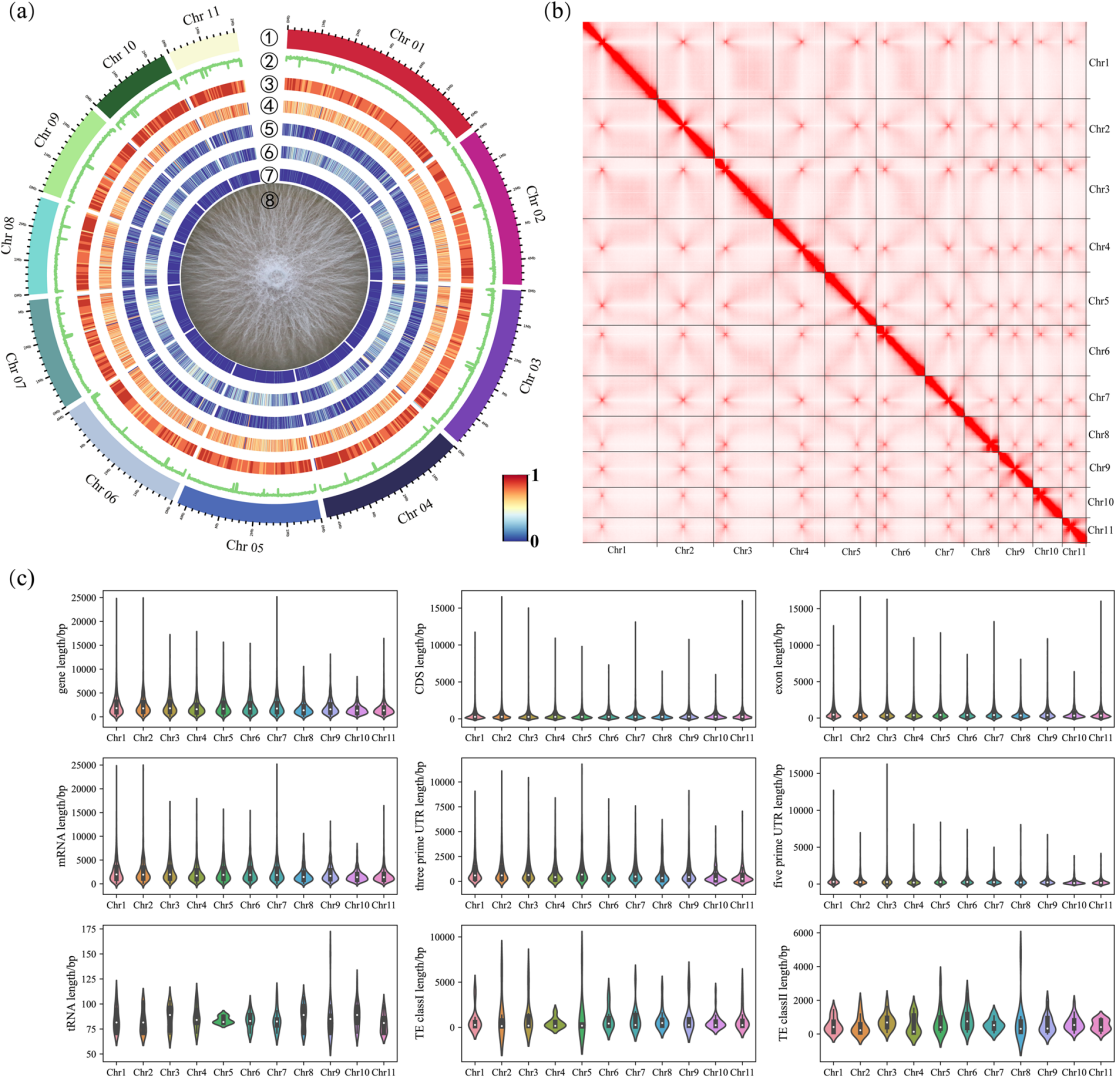
Overview of the gap-free reference genome and annotation of *Fusarium verticillioides* strain 7600. (**a**) Circos plot showing the gene features at 10 kb windows across the 11 chromosomes in *F. verticillioides* strain 7600. From outer to inner ring: ① chromosome ideogram, ② GC content, ③ gene density, ④ exon density, ⑤ TE (transposable element) density, ⑥ Simple repeat density, ⑦ t-RNA density, ⑧ Colony morphology photographed after 6-day incubation at 25 °C. (**b**) High-throughput chromatin conformation capture (Hi-C) interaction map of *F. verticillioides* strain 7600 visualizes the number of chromosome interactions within and between 11 chromosomes. (**c**) Violin plots of genomic features, including gene length, CDS length, exon length, mRNA length, three prime UTR (untranslated region), five prime UTR, tRNA length, TE class I, TE class II.

Here, we aim to produce a gap-free reference genome of *F. verticillioides*, and update the genome annotations based on the improved genome assembly. We sequenced the genome of *F. verticillioides* strain 7600 to produce high-fidelity (HiFi) long reads of PacBio (Pacific Biosciences, CA) single-molecule real-time (SMRT) sequencing, and Hi-C (high throughput chromatin conformation capture) data using Illumina pair-end sequencing. In total, we generated 4.1 Gb (~96.7X coverage) PacBio HiFi raw reads with a N50 of 10.0 kb. and 53.8 Gb Hi-C data (Illumina paired-end reads, ~1,272X coverage) (Table 1). For genome assembly, HiFi data were assembled using multiple tools including Hifiasm^5^, HiCanu^6^, NextDenovo (https://github.com/Nextomics/NextDenovo) and Flye^7^ to obtain draft genome assemblies which were individually polished using Nextpolish (v.1.4.0)^8^ followed by assembly merge using quickmerge (https://github.com/mahulchak/quickmerge) (Table 2). Then, Hi-C data were used to anchor the contigs onto chromosomes using Juicer^9^ and 3d-DNA pipeline^10^. The final genome assembly (42.0 Mb) contains 11 gap-free chromosomes (Figure 1a,b) with a contig N50 of 4.3 Mb, a significant improvement (+989.5%) compared to the previous version GCA_000149555.1 (contig N50 = 392.4 kb) (Table 3).

**Table 1:** A summary of sequencing data output of *Fusarium verticillioides* strain 7600 for genome assembly and annotation.

Statistics	PacBio HiFi	Hi-C	RNA-seq
Library size (bp)	15,000	350	350
Raw data (Gb)	4.1	53.8	10.5
N50 (bp)	10,027	150	150
Longest reads (bp)	32,687	150	150
Mean read length (bp)	9,196.90	150	150
Coverage (X)	96.7	1272.8	N/A

**Table 2:** Genome assembly statistics for different assemblers and their merged results using quickmerge.

Assembler	Assembly Length (Mb)	No. of contigs	Longest Contig (Mb)	Contig N50 (Mb)
Hicanu	44.883	162	6.253	4.275
Flye	42.346	16	6.253	4.275
HiFiasm	43.981	81	6.253	4.275
NextDenovo	42.352	13	6.253	4.275
NextDenovo + Flye	42.359	13	6.253	4.275
NextDenovo + Flye + HiFiasm	42.374	13	6.253	4.275
NextDenovo + Flye + HiFiasm + Hicanu	42.374	13	6.253	4.275

**Table 3:** Genome assembly and annotation statistics.

Statistics		GCA_000149555.1	This Study	Difference (±%)
Assembly	Assembly Size (bp)	41,791,161	41,994,356	0.5
	Number of contigs	211	11	−94.8
	Contig N50 (bp)	392,397	4,275,051	989.5
	Contig N90 (bp)	112,447	2,453,640	2082
	Number of Scaffolds	22	11	−50
	Scaffold N50 (bp)	4,236,349	4,275,051	0.9
	Scaffold N90 (bp)	3,901,718	2,453,640	−37.1
	Longest scaffold (bp)	6,219,215	6,252,867	0.5
	Gap bases (bp)	90,816	0	−100
Annotation	Number of genes	14,335	15,230	6.20%
	GC Content (%)	48.6	48.3	−0.3
	Retroelements (bp)	65,239	184,874	183.4
	SINEs (bp)	5,952	10,343	73.8
	LINEs (bp)	0	54,265	54,265
	LTR elements (bp)	59,287	120,266	102.9
	DNA transposons (bp)	3,790	102,640	2608.2
	Unclassified TEs (bp)	281,241	441,282	56.9
	Simple repeats (bp)	250,522	264,291	5.5
	Low complexity (bp)	31,817	34,029	7
Quality Assessment	BUSCO (%)	99.9	99.9	0
	Quality value	42.4	88.8	109.4
	CEGMA (%)	99.1	99.6	0.5
	NGS mapping ratio (%)	98.2	98.3	0.1

For the gap-free assembly, we performed genome annotation to predict protein-coding genes and repeat elements. To see how much a nearly complete genome sequence improves genome annotations, the same annotation pipeline and RNA-seq data were applied to annotation of both our assembly and the previous version GCA_000149555.1. For protein-coding genes, the two genome assemblies were comparable where our assembly encodes 15,230 genes, a slight increase (+6.2%) compared to the previous assembly (Table 3; Fig. 1c). Comparing the two annotations revealed 15,056 genes present in both genome assemblies while 75 and 174 genes were uniquely annotated using previous and our genome assembly, respectively. The new genome assembly contains 2.8% (1,164,494 bp) repeat content, higher than the previous version (1.7%, 708,545 bp). Specifically, our assembly contains 120,266 bp LTR (long terminal repeat) element (+102.9%) and 102,640 bp DNA transposon (+2,608.2%) (Table 3).

**Fig. 2:**
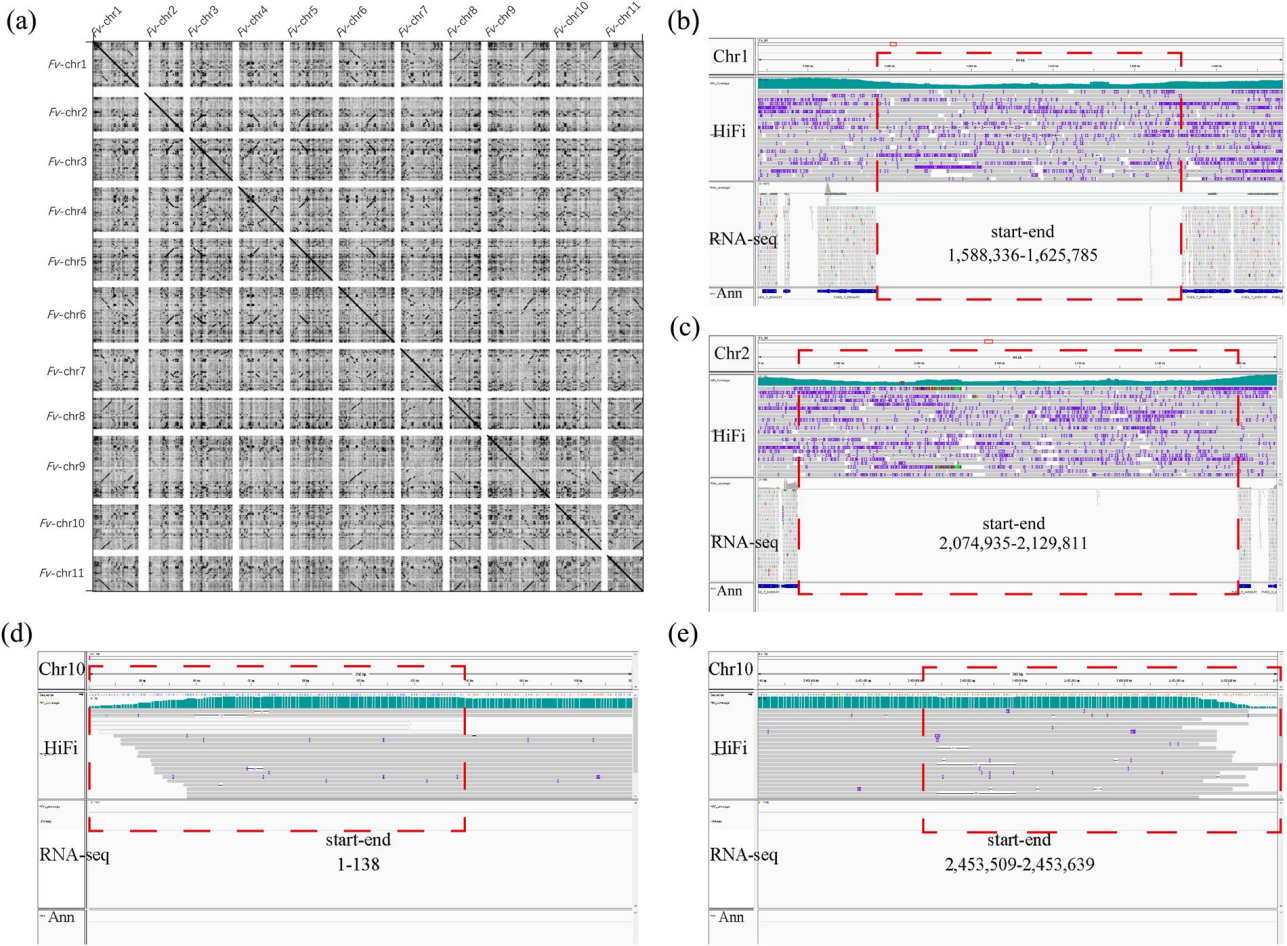
Features and validation of telomeres and centromeres of *Fusarium verticillioides* strain 7600. (**a**) Dotplot of *F. verticillioides* centromere sequences assembled in this study visualized using GePard. (**b**,**c**) IGV (integrative genomics viewer) visualization of centromere regions (dashed red box) where PacBio HiFi and RNA-seq reads are mapped, from chromosomes 1 and 2, respectively. (**d**,**e**) IGV visualization of two telomere regions of chromosome 10 where PacBio HiFi and RNA-seq reads are mapped.

**Fig. 3:**
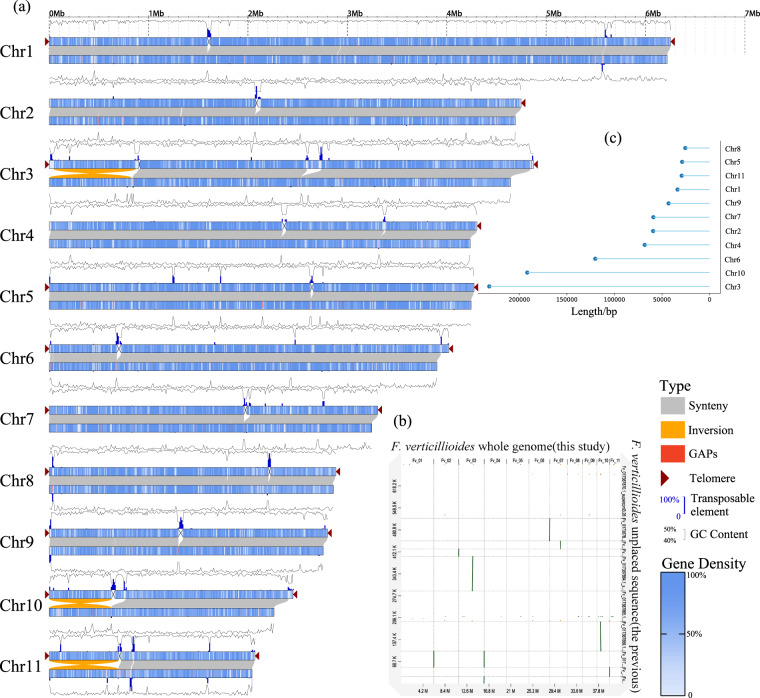
*Fusarium verticillioides* strain 7600 gap-free genome assembly represents a major improvement over the previous version. (**a**) Comparison of *F. verticillioides* genome characteristics between the previous version (GCA_000149555.1) and the gap-free assembly in this study. (**b**) Dotplot displaying the alignment of unplaced sequence of the previous genome against the gap-free chromosomes assembled in this study, indicating successful chromosome anchor of these sequences. (**c**) Lollipop plot summarizing the length of newly assembled sequences per chromosome compared to the previous version of the genome.

Compared to previous genome assemblies, this gap-free genome assembly of *F. verticillioides* contained all centromeres on 11 chromosomes (Fig. 2a), thanks to the highly accurate HiFi sequence data and improved assembly algorithms. To validate the centromere regions, we mapped the HiFi reads and RNA-seq reads to the gap-free assembly. We found a decent coverage of HiFi reads throughout the assembly including the centromeres (Figure 2b,c) and telomeres (Figure 2d,e) which contained no protein-coding genes and little RNA-seq alignment. By comparing this assembly with a previous assembly (GCA_000149555.1), we showed that numerous gaps were closed and three large inversions on the short arms of Chr3, Chr10 and Chr11 were corrected in this new assembly (Fig. 3a). Furthermore, unplaced scaffolds in GCA_000149555.1 are now anchored to correct chromosome positions (Fig. 3b). The gapless assembly contained a total of 890 kb new sequences including 25 kb to 231 kb per chromosomes which were absent in GCA_000149555.1 chromosomes (Fig. 3c).

**Fig. 4:**
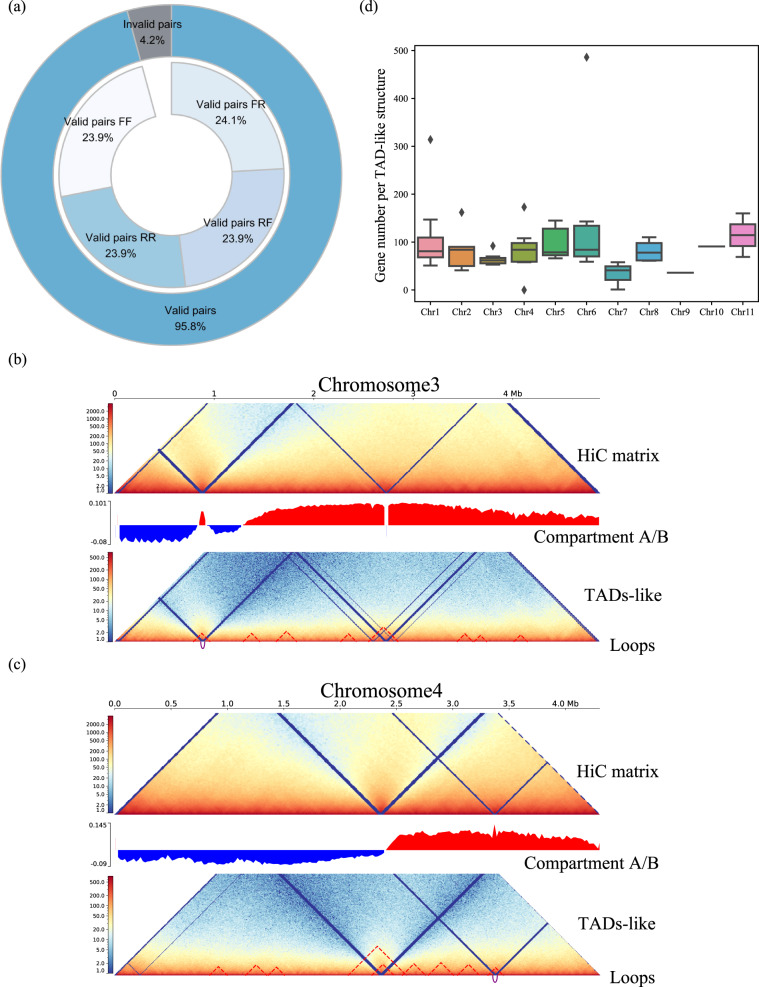
Characteristics of the three-dimensional genome of *Fusarium verticillioides* strain 7600. (**a**) Donut chart summarizing the results of Hi-C data quality control performed by HiC-Pro. (**b,c**) Three-dimensional genomic feature (Hi-C matrix, A/B compartment, TADs(topological associated domains)-like structures and chromatin loops) for chromosome 3 and 4. (**d**) Boxplot summarizing the number of genes co-localized within TADs-like regions on each chromosome.

Lastly, we analyzed the three-dimensional genome of *F. verticillioides* based on the Hi-C sequencing data, generated from fungal mycelia collected from culture. With a total of 53.8 Gb (1272.8X coverage) Hi-C data containing 95.8% valid interaction pairs after initial quality control (Fig. 4a), from which we identified 60 TADs (topological associated domains) -like structures and five chromosome loops under 10 kb resolution (Table 4; Supplementary Table 1; Fig. 4b,c). Various candidate protein-coding genes were localized within the TADs-like and loop structures (Table 4; Supplementary Table 1; Fig. 4d). This gap-free genome assembly and updated annotation of *F. verticillioides* are excellent resources to study mechanisms of fungal genome evolution, mycotoxin production and pathogenesis on plant and human host.

**Table 4:** Genomic coordinates of chromatin loops in *Fusarium verticillioides* genome.

Chromosome ID	X1	X2	Y1	Y2
Chr3	865,000	870,000	900,000	905,000
Chr4	3,355,000	3,360,000	3,390,000	3,395,000
Chr8	2,195,000	2,200,000	2,230,000	2,235,000
Chr10	740,000	745,000	775,000	780,000
Chr11	690,000	695,000	725,000	730,000

X and Y represent the corresponding regions connected by a chromatin loop structure, where 1 and 2 mark the start and end of the region, respectively.

## Methods

### Fungal culture, DNA preparation and PacBio HiFi sequencing

*F. verticillioides* strain 7600 was routinely maintained on PDA (potato dextrose agar) slant and stored in −80 °C freezer. *F. verticillioides* 7600 mycelia and spores harvested from two-day old PDB (potato dextrose broth) culture in 150 rpm shaker at 25 °C were used to isolate high molecular weight DNA using CTAB (cetyltrimethylammonium bromide) method^11^. A total of 15 µg purified genomic DNA were used to construct a standard PacBio SMRTbell library using PacBio SMRT Express Template Prep Kit 2.0 (Pacific Biosciences, CA). The sequencing was performed using a PacBio Sequel II instrument at Biomarker Technologies Corporation (QingDao, China).

### Hi-C sequencing and analysis

Hi-C library construction of *F. verticillioides* was prepared from cross-linked chromatins of fungal mycelia using a standard Hi-C protocol^12^. The constructed Hi-C sequencing library was sequenced by a test run and examined for valid interaction read pair ratios using HiCPro (v.3.1.0)^13^ before going through high coverage sequencing. The library was sequenced by Illumina NovaSeq. 6000 to yield 10.5 Gb (249.7 coverage) paired-end reads. The valid interaction pairs of Hi-C sequencing reads were used to anchor all contigs using Juicer (v.1.5)^9^, followed by using a 3D-DNA correction pipeline^10^ and manually correction with Juicebox (v.1.11.08)^14^. compartment A/B were analyzed using HiTC (v.1.40.1)^15^ and Cworld-dekker (https://github.com/dekkerlab/cworld-dekker), TADs-like structures and chromosome loop were identified by Juicer (v.1.5)^9^. Three-dimensional structure visualization of the whole genome using pyGenomeTracks (v.3.7)^16^.

### Genome assembly

To optimize the genome assembly strategy and take into account the differences of assembly algorithm between software, we used Hifiasm (v.0.16.1)^5^, HiCanu (v.1.4)^6^ (parameters: -assemble -pacbio-hifi oeaErrorRate = 0.001), Flye (v.2.9)^7^ and NextDenovo (v.2.5.0) (https://github.com/Nextomics/NextDenovo, with parameters: minimap2_options_cns = -x ava-hifi), to assemble, respectively, and then sorted the number of contigs of different assemblers in ascending order. Based on four assemblies we used quickmerge (v.0.3) (https://github.com/mahulchak/quickmerge) to produce a merged genome assembly, and finally used Juicebox^14^ to manually adjust misassemblies.

### RNA sequencing and analysis

Total RNA was extracted from the mycelia of *F. verticillioides* using Trizol (Thermal Fisher) agents following manufacturer recommendation protocol. The RNA Nano 6000 Assay Kit of Agilent Bioanalyzer 2100 system (Agilent Technologies, CA, USA) was used to evaluate the total RNA integrity. The total RNA used for library preparation first enriched the mRNA with polyA tail through Oligo (dT) magnetic beads. The mRNA was then subjected to sequencing library construction using Illumina True-seq transcriptome kit (Illumina, CA) with an insert size of 370bp–420bp, and sequenced by an Illumina Novaseq. 6000 platform at Biomarker Technologies Corporation (QingDao, China) to generate 150 bp paired-end reads. RNA-seq data was checked for quality using fastp (v.0.23.2)^17^, mapped to *F. verticillioides* genome assembly using hisat2 (v.2.1.0)^18^, followed by calculating mapping ratios by samtools (v.1.15)^19^.

### Identification of gene model and prediction of repeat sequences and non-coding RNA

For repetitive sequences, we firstly use *de novo* prediction and similarity aligmment to annotate it via RepeatModeler (v. 1.0.11)^20^ (parameters: -database -engine ncbi -pa) and softmasked genome by RepeatMasker (v. 4.1.2.p1)^21^. RepeatMasker’s perl script (rmOutToGFF3.pl) converts various types of repeat sequence annotation results into a common generic feature format (GFF) version 3. Gene model prediction combined with the following three aspects of evidence: (a) *ab initio* prediction, (b) homologous protein, (c)RNA-seq evidence. During the *ab initio* prediction, we firstly trained the GeneMark-ET model for five rounds using BRAKER2 (v.2.1.6)^22^ (parameters:–species = Fv–fungus–softmasking–genome–bam–prot_seq–prg = gth–gff3–rounds = 5), whose process employed GeneMark-ET^23^, NCBI BLAST^24^, DIAMOND^25^ and GenomeThreader^26^. We then trained the SNAP^27^ semi-HMM model for two rounds using MAKER^28^ (parameters: est2genome = 1, protein2genome = 1, pred_flank = 100, alt_splice = 1, correct_est_fusion = 1). AUGUSTUS^29^ used the built-in *Fusarium* genome feature model. To provide homologous protein evidences for gene prediction, we downloaded the protein data of this species (anchored chromosomes) from the public database, including 7600 (NCBI Assembly ID: GCA_ 000149555.1), BRIP53590 (NCBI Assembly ID: GCA_003316995.2), BRIP53590 (NCBI Assembly ID: GCA_003317015.2) and BRIP14953 (NCBI Assembly ID: GCA_003316975.2). For transcriptome data, RNA-seq reads from the vegetative phase were firstly aligned to our genome assembly through hisat2^18^ for BRAKER2 (v.2.1.6)^22^. Then, we performed reference-based assembly and *de novo* assembly of transcriptomes by Scallop (v0.10.5)^30^ and Trinity (v.2.8.4)^31^ (parameters:–min_kmer_cov 3–normalize_max_read_cov 100), respectively. Transcripts obtained by two methods are de-redundant with CD-HIT (v.4.6)^32^ (parameters:-I -c 0.99 -T 50 -M 100000 -o). The above three evidences are integrated by MAKER (v.3.01.03)^28^ to predict the final gene model. Rfam/Infernal (v.1.1.4)^33^ (parameters: cmscan–cut_ga–rfam–nohmmonly–fmt 2 –clanin –tblout) and tRNAscan-SE (v. 2.0.9)^34^ (parameters: -E -X 20 –f -m -b -j–detail) are used to infer genome-wide non-coding RNAs. To compare the previous (NCBI: GCA_000149555.1) and our genome assembly, we performed the genome annotation on two genome assemblies by using the same software, parameters, and protein data.

## Data Records

The raw PacBio HiFi sequencing data, Hi-C data and RNA-seq data have been deposited in the National Center for Biotechnology Information (NCBI) under the BioProject (PRJNA868307)^35^ with accession number of SRR21003521^36^, SRR21003520^37^, SRR21003519^38^, respectively. The gap-free genome assembly is deposited under the same BioProject at NCBI (GCA_027571605.1) and also in Genome Warehouse of National Genomics Data Center (https://ngdc.cncb.ac.cn/) at China National Center for Bioinformation under the accession number of GWHBQEB00000000^39^. Genome annotations including protein-coding regions, repeat sequence and ncRNA annotation files have been submitted to the online open access repository Figshare^40^.

## Technical Validation

### Manual adjustment of misjoin and detection of potentially contaminated sequences

To get a nearly complete and error-free nuclear genome, we first manually corrected the assembly using Hi-C read alignment within the Juicebox^14^. We then aligned the species’ mitochondrial genome to our assembly by megaBLAST^24^, which found no errors. Finally, we also used megaBLAST^24^ to aligned our genome assembly against a common database (ftp://ftp.ncbi.nlm.nih.gov/pub/kitts/contam_in_euks.fa.gz) to identify potentially contaminated sequences sequencing adaptor sequence (ftp://ftp.ncbi.nlm.nih.gov/pub/kitts/adaptors_for_screening_euks.fa) and nucleotide sequence database (remote mode), which again found no contamination.

### Evaluation of the genome assembly

The genome assembly was validated by two independent methods. Firstly, HiFi reads were mapped to the assembly using Winnowmap2 (v.2.03)^41^ (parameters: -W repetitive_k15.txt -t 104 -ax map-pb) and the quality value (QV) was assessed using Merqury (v.1.3)^42^ (parameters: k = 18 count). Second, the BUSCO (Benchmarking Universal Single-Copy Ortholog)^43^ analysis was conducted to reflect the completeness of genome assembly. The final *F. verticillioies* gap-free genome assembly has a QV of 88.8, completeness of 99.7% and BUSCO score of 99%, suggesting the high accuracy and completeness of the assembly, respectively (Table 2).

### Validation of the genome assembly

The resolved fungal telomere and centromere regions have been well covered by PacBio HiFi reads that span these complex regions (Fig. 2) by IGV (v.2.4.10)^44^. This assembly has reduced the length of gaps from 90,816 in previous version to 0, and captured eleven centromeres and nineteen telomeres (TTAGGG) except missing three telomeres via trf (v. 4.09.1)^45^ (parameters: 2 7 7 80 10 90 2000 -d -m -l 2) from assemblies and raw sequences, one each at the end of Chr2 and Chr4 (Fig. 3). There is a one-to-one correspondence between the old and new versions of the genome with 14,260 coding region genes via liftoff (v.1.6.3)^46^ and BEDtools (v.2.30.0)^47^ (parameters: intersect -wa -wb -f 1.0), which account for 99.5% of the old version genome and 93.6% of this study genome. Compared to previous version (NCBI: GCA_000149555.1), our assembly has corrected three major inversions (Fig. 3) located at the short arm of Chr3, Chr10 and Chr11 visualized via GenomeSyn^48^ plot.

## Supplementary information

Supplementary Table 1

## Data Availability

All software used in this study are in the public domain, with parameters being clearly described in Methods and this section. If no detail parameters were mentioned for the software, default parameters were used as suggested by developer.
